# A C-shaped hinge for displacement magnification in MEMS rotational structures

**DOI:** 10.1038/s41378-023-00618-9

**Published:** 2024-01-04

**Authors:** Naga Manikanta Kommanaboina, Teferi Sitotaw Yallew, Alvise Bagolini, Maria F. Pantano

**Affiliations:** 1 Department of Civil, Environmental and Mechanical Engineering, University of Trento, via Mesiano 77, 38123 Trento, Italy; 2 Fondazione Bruno Kessler (FBK), Microsystems Technology (MST), via S. Croce 77, 38122 Trento, Italy

**Keywords:** NEMS, Engineering

## Abstract

The design, analysis, fabrication, and characterization of two distinct MEMS rotational structures are provided; these structures include a classical symmetrical lancet structure and a novel symmetrical C-shaped structure provided with a tilted arm, and both are actuated by thermal actuators. Our proposed C-shaped structure implemented a curved beam mechanism to enhance the movement delivered by the thermal actuators. The geometrical parameters of our proposed device were optimized using the design of experiment (DOE) method. Furthermore, the analytical modeling based on Castigliano’s second theorem and the simulations based on the finite element method (FEM) were used to predict the behavior of the symmetrical C-shaped structure; the results were in good agreement with each other. The MEMS-based rotational structures were fabricated on silicon-on-insulator (SOI) wafers using bulk micromachining technology and deep reactive ion etching (DRIE) processes. The fabricated devices underwent experimental characterization; our results showed that our proposed MEMS rotational structure exhibited a 28% improvement in the delivered displacement compared to the symmetrical lancet structure. Furthermore, the experimental results showed good agreement with those obtained from numerical analysis. Our proposed structures have potential applications in a variety of MEMS devices, including accelerometers, gyroscopes, and resonators, due to their ability to maximize displacement and thus enhance sensitivity.

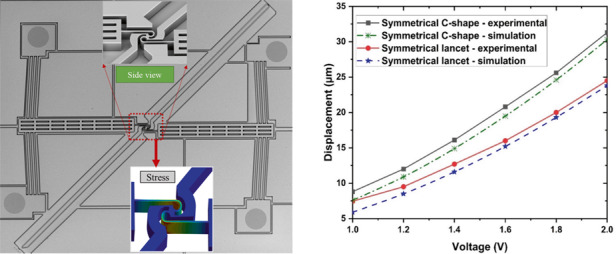

## Introduction

Microelectromechanical systems (MEMS) have revolutionized the field of sensors, actuators, energy harvesters, switches, and microgrippers by enabling the integration of mechanical and electrical components on a microscale^1^. Over the years, researchers have made significant strides in improving the precision and size of MEMS devices. In common MEMS actuators, electrical signals are converted into mechanical displacement, leading to a change in capacitance or resistance. However, these changes are often small and require amplification mechanisms to improve the sensitivity of the device^2,3^. Amplifying displacement is a critical aspect of MEMS technology, especially in applications where a large movement or change in capacitance is needed^4^. Different mechanisms have been proposed^1,3,5^ to amplify the displacements in the actuators, sensors, and stress diagnosis structures. In particular, producing high displacement in microactuators has been challenging and highly important in the field of MEMS. The enhancement of microactuator performance can be achieved through the integration of an intermediate mechanism that can amplify the delivered displacement at the output. These miniaturized amplification mechanisms exhibit low power consumption, are light weight, and can be easily manufactured through standard MEMS fabrication techniques^1^. Flexure-based compliant mechanisms are showing growing potential in the field of precision engineering, robotics, microgrippers, and various other applications owing to their exceptional benefits in addressing issues, such as friction, backlash, and wear in conventional precision systems^6^. In general, flexure-based mechanisms can be categorized into two types: planar mechanisms and 3D mechanisms^7^. However, planar flexure-based mechanisms have gained the most widespread applications. These planar mechanisms possess monolithically machined structures, enabling them to achieve precise motion control. They usually consist of single-axis flexure hinges^8^, such as circular, corner-filled, elliptical, and constant rectangular cross-section flexure hinges, which facilitate two-dimensional motion. Different designs of flexure-based planar compliant mechanisms for motion amplification are reported in^9,10^. Furthermore, a more recent development in the field is the conjugate surface flexure hinge (CSFH)^11^, which can be constructed as a single entity and easily incorporated into any MEMS mechanical structure. The CSFH provides several benefits, including the reduction of internal stress, its durability during operation, and the optimization of the overall relative hinge rotations. These hinges are implemented to construct planar, compliant, and monolithic microgrippers, supporting rotational motion, for cell and tissue manipulations^12–14^.

MEMS-based rotational structures, such as lancet-based structures^5,15^, are also widely used to measure stress, which is a crucial factor in optimizing residual stresses left by microfabrication processes. One of the main advantages of rotational structures is their ability to measure both tensile and compressive stresses with a continuous readout. This feature is important because it enables the characterization of the stress distribution in real time. Currently, the MEMS-based fabrication technology used in rotational structures enables them to occupy a limited area on the wafer; thus, they are more suitable for high-density integration. Furthermore, rotational structures have the ability to directly amplify displacement with consequent sensitivity enhancement, causing them to be more suitable for stress measurements in small devices^16,17^. However, despite their advantages, rotational structures also have certain limitations. For instance, they may not achieve a large displacement with a smaller input force and are more vulnerable to breakage due to localized stress^5^.

Typically, rotational structures have been developed using two main fabrication techniques: surface micromachining and bulk micromachining^5,18^. Surface micromachining is a well-established process for the fabrication of MEMS structures using thin films mainly produced by low-pressure chemical vapor deposition. The films are etched and patterned to form the desired device structure. However, the residual stress in these thin films can be high, leading to buckling or cracking of the structures upon released. To mitigate this issue, various stress-reducing techniques have been developed, such as the use of multilayer thin films, postdeposition annealing, and the introduction of stress-relief structures^18^. On the other hand, bulk micromachining involves the deep etching of the substrate material (usually silicon) to form the desired device structures. This method has advantages over surface micromachining. First, it leverages the properties of single-crystal silicon to produce structures that are more robust and stable compared to thin films used in surface micromachining. Thin films are more prone to deformations and long-term stability issues. Second, bulk micromachining utilizes anisotropic etching processes to create oriented structures with 3D dimensions and shapes. Furthermore, the mechanical superiority of single-crystal silicon contributes to the appeal of micromachining since it provides stiffness, strength, and wear resistance; all of these are crucial qualities for MEMS devices with rotational components that require robust mechanical integrity. Additionally, bulk micromachining helps overcome challenges related to stress during fabrication when compared to surface micromachining, which involves the film deposition and release procedures introducing significant residual stresses that can negatively affect the performance. The choice between surface and bulk micromachining depends on the requirements of the application at hand. While surface micromachining provides versatility and cost effectiveness, bulk micromachining is better suited for applications emphasizing reliability, stability, and intricate three-dimensional structures^19,20^.

In this study, we developed a novel MEMS-based rotational structure, embedding C-shaped symmetrical hinges to provide displacement magnification capability; these were fabricated via a bulk micromachining process with silicon-on-insulator (SOI) wafers. The SOI wafers consisted of a single-crystal silicon layer on top, an insulating layer, and a handle layer on the bottom^21^. In the past, the use of SOI wafers showed several advantages over traditional silicon bulk substrates. First, SOI wafers exhibited lower residual stresses in the structural layer, resulting in higher quality and reliability. Second, the insulating layer provided an excellent etch stop, simplifying the fabrication process. Third, the isolation of the single-crystal layer from the bulk substrate led to lower parasitic capacitances, enabling high-speed devices with lower power consumption. Finally, the complete isolation of n-well and p-well devices in the SOI wafers provided protection against latch-up effects and enhanced the radiation hardness^22^. Leveraging the advantages provided by SOI wafers, our novel rotational structure was mechanically robust. Finally, with the purpose of quantitatively evaluating the advantages provided by our proposed novel design, we considered the rotating lancet structure as a well-assessed baseline to compare its classical design performance with the improved performance of our proposed novel rotational hinge.

## Materials and methods

### Parameter optimization method

Design of Experiment (DOE) is a statistical method used to identify the relationship between the independent variables (factors) and the dependent variables (responses or outputs) of a process or system. It allows for systematic investigation and optimization of complex systems, processes, or designs. DOE helps to determine the most significant factors affecting the output performance, as well as their interactions, and optimizes the system for maximum efficiency and effectiveness. Different methods exist, such as one-factor, factorial design, robust parameter design, and reliability design, providing different approaches and levels of detail to analyze and improve the system output response^23,24^.

The design parameters of our novel MEMS rotational structure were optimized using the response surface method (RSM) in combination with central composite design (CCD) run via JMP® statistical software. RSM is a widely used multivariate technique for modeling the relationship between a dependent variable (response) and multiple independent variables^24,25^. In this study, we investigated the effect of two parameters characterizing the geometry of the C-shaped hinge: the curved beam width (W) and the gap (K) between the upper and lower curved beam centers of rotation; these were the independent variables affecting the response of the system, i.e., the displacement at the tip of the tilted arm. To determine the optimal values for the independent variables, three levels were selected for each factor: low (−), medium (0), and high (+). Based on fabrication limits, the low and high levels for W were established at 2.3 µm and 6 µm, respectively. Then, an intermediate level of 4.5 µm was also considered. Similarly, the low and high levels for K were set at 15 µm and 30 µm, respectively. An intermediate level at 22.5 µm was also considered (Table 1). For each combination of W and K, we performed finite element method (FEM) -based 3D thermal-electric-structural simulations in the Ansys software to determine the corresponding displacement produced at the tip of the tilted arm. The use of RSM in conjunction with CCD enabled a comprehensive investigation of the effect of the independent variables on the response and the determination of the optimal design parameters for the structures.

**Table 1:** Design (CCD) matrix identified to optimize the geometry of the C-shaped rotational structure

Observations	Treatments	Factor W (µm)	Factor K (µm)	Displacement (µm) at 1 V
1	− −	2.3	15	6.2
2	+ −	6	15	2.1
3	− +	2.3	30	5.31
4	+ +	6	30	2.62
5	0 −	4.5	15	3.56
6	0 +	4.5	30	3.72
7	− 0	2.3	22.5	5.94
8	+ 0	6	22.5	2.44

The matrix reports different values of the input variables (curved beam width, W, and gap between the upper and lower curved beam centers of rotation, K) and the corresponding system output response (displacement at the tip of the tilted arm), determined via FEM-based simulations

The overall variation in the output response of each factor can be evaluated by calculating the total sources of variance, denoted as *SS*_*T*_, which consists of two components: factor sum of squares (*SS*_*A*_), representing the effects of the factors, and error sum of squares (*SS*_*E*_), representing random error. These values can be determined using the following formulas^24^:

1 $${{SS}}_{T}={{SS}}_{A}+{{SS}}_{E}$$

2 $${{SS}}_{A}=\mathop{\sum }\limits_{i=1}^{a}\mathop{\sum }\limits_{j=1}^{{n}_{i}}({{\bar{y}}_{{ij}}-\bar{y})}^{2}$$

3 $${{SS}}_{E}=\mathop{\sum }\limits_{i=1}^{a}\mathop{\sum }\limits_{j=1}^{{n}_{i}}({{y}_{{ij}}-\bar{{y}_{{ij}}})}^{2}$$

where ${\bar{y}}_{{ij}}$ is the factor level group mean, $\bar{y}$ is the overall mean, *a* is the number of levels of the factor, *y*_*ij*_ is the jth response in the ith factor level and *n*_*i*_ is the number for which the factor is at level *i*.

To determine the significant factors influencing the output response, an F test based on ANOVA (analysis of variance) was performed. This test investigates the following hypothesis for each factor (µ_*i*_):

$${H}_{0}\!:{\mu }_{1}={\mu }_{2}=\ldots ={\mu }_{a}$$

*H*_1_: *μ*_*i*_ ≠ *μ*_*j*_ for at least one pair (*i,j*)

The *F* value (*F*_0_) can be obtained by:

4 $${F}_{0}=\frac{{{SS}}_{A}/a-1}{{{SS}}_{A}/N-a}=\frac{{{MS}}_{A}}{{{MS}}_{E}}$$

where (*a* − 1) is the degrees of freedom for factor A, (*N* − *a*) is the error degrees of freedom, *MS*_*A*_ and *MS*_*E*_ are the mean sum of squares and the error sum of squares for factor A, respectively. If *F*_0_ is higher than the threshold value ${F}_{{\rm{\alpha }},{\rm{a}}-1,{\rm{N}}-{\rm{a}}}$, where α is the level of significance, the null hypothesis is rejected. The analysis of variance for the two factors is presented in Table 2 along with the corresponding F and *p* values. The relevant factors impacting the output response are screened out at the 95% level of significance (*p-value* < 0.05)^24^.

**Table 2:** Effect test results from the JMP® Statistical Software

Varying factor	Degree of freedom	Sum of squares	*F* value (*F*_0_)	*P*-value (<0.05)	Significance
W	1	17.647350	814.1799	0.0012	Yes
K	1	0.007350	0.3391	0.6193	No
W: K	1	0.497025	22.9308	0.0409	Yes
W: W	1	0.232408	10.7224	0.0820	No
K: K	1	0.023408	1.0800	0.4078	No

### Analytical model

Due to the symmetry of the C-shaped rotational structure (Supporting Fig. S1a), we can model its behavior by considering the simplified structure shown in Supporting Fig. S1b, c. This consists of a straight beam (AB) with length *x*_*s*_, connected to a curved beam (BC) with radius R, which is in turn connected to a rigid element DC with length *x*_*r*_. Regarding the boundary conditions, we can assume the presence of a slider at A, which accounts for the presence of the thermal actuator delivering a horizontal displacement and force (*F*_*A*_), and a hinge at D since the structure can freely rotate around it.

We used Castigliano’s second theorem to calculate the horizontal displacement (*δ*_*A*_) of the straight beam (i.e., input to the system) as a function of the force delivered by the thermal actuator (*F*_*A*_)^26,27^. Based on this theorem regarding displacements in a linearly elastic structure, if the strain energy of a linearly elastic structure can be expressed as a function of generalized force, *F*_*i*_, then the generalized displacement *δ*_*i*_ in the direction of *F*_*i*_ can be computed as the partial derivative of the total strain energy, *U*_*total*_, with respect to generalized force, *F*_*i*_^28^, which in our case provides the following:

5 $${\delta }_{A}=\frac{\partial {U}_{{total}}}{\partial {F}_{A}}$$

The total strain energy can be determined as follows^29^:

6 $${U}_{{total}}={U}_{{straight\,beam}}+{U}_{{curved\,beam}}={\int }_{0}^{{x}_{s}}\frac{{M}^{2}\left(x\right)}{2{EI}}{dx}+{\int }_{0}^{\pi +{\theta }_{1}}\frac{{M}^{2}\left(\alpha \right)}{2{EI}}{Rda}$$

where *I* is the moment of inertia of the cross-section (i.e., $I=\frac{{W}^{3}h}{12}$, where h is the thickness) and *E* is Young’s modulus. By considering Supporting Fig. S1b, c, the moment along the straight *M*(*x*) and curved *M*(*α*) beams can be written as follows:

7 $${\rm{M}}\left(x\right)={{\rm{R}}}_{{\rm{A}}}\,{\cdot\, x}+{\rm{Y}}$$

8 $${\rm{M}}\left(\alpha \right)=-{F}_{A}{\rm{R}}\left(1-\cos \alpha \right)+{R}_{A}({x}_{s}+{R}\sin \alpha )+{\rm{Y}}$$

where R_A_ and Y are the vertical reaction force and the moment provided by the slider at A, respectively.

R_A_ can be computed as ${R}_{A}={R}_{A}^{(0)}-Y{R}_{A}^{(1)}$, where ${R}_{A}^{\left(0\right)}$ ${=}\frac{{F}_{A}R\left(1+\cos {{{\theta}}}_{1}\right)}{{x}_{s}-\left({x}_{r}+{R}\sin {{{\theta}}}_{1}\right)}$ and ${R}_{A}^{\left(1\right)}=\frac{1}{{x}_{s}-\left({x}_{r}+{R}\sin {{{\theta}}}_{1}\right)}$ and Y can be computed by the virtual work method as follows:

$${\rm{Y}}=-\left(\frac{{\left[-{R}_{A}^{\left(0\right)}{R}_{A}^{\left(1\right)}\left(\frac{{z}^{3}}{3}\right)+{R}_{A}^{\left(0\right)}\left(\frac{{z}^{2}}{2}\right)\right]}_{z=0}^{{z=x}_{s}}+{\left[-{R}_{A}^{\left(1\right)}{FR}^{3}\cos \alpha -{R}_{A}^{\left(1\right)}{FR}^{3}\left(\frac{{\sin }^{2}\alpha }{2}\right)+{R}_{A}^{\left(1\right)}{FR}^2{x}_{s}\alpha -{R}_{A}^{\left(1\right)}{FR}^2{x}_{s}\sin \alpha -{FR}^2\alpha +{FR}^2\sin \alpha -{R}_{A}^{\left(0\right)}{R}_{A}^{\left(1\right)}{R}^{3}\left(\frac{\alpha }{2}-\frac{\sin 2\alpha }{4}\right) -{R}_{A}^{\left(0\right)}R^2\cos \alpha -{R}_{A}^{\left(0\right)}{R}_{A}^{\left(1\right)}{{x}_{s}}^{2}R\alpha +2{R}_{A}^{\left(0\right)}{R}_{A}^{\left(1\right)}R^2{x}_{s}\cos \alpha +{R}_{A}^{\left(0\right)}{x}_{s}\alpha\right]}_{\alpha =0}^{\alpha =\pi +{\theta }_{1}}}{{\left[{{R}_{A}^{\left(1\right)}}^{2}\left(\frac{{z}^{3}}{3}\right)-2{R}_{A}^{\left(1\right)}\left(\frac{{z}^{2}}{2}\right)+z\right]}_{z=0}^{z={x}_{s}}+{\left[{{R}_{A}^{\left(1\right)}}^{2}{Rx}^{2}\alpha -2{{R}_{A}^{\left(1\right)}}^{2}{R^2}x\cos \alpha +{{R}_{A}^{\left(1\right)}}^{2}{R}^{3}\left(\frac{\alpha }{2}-\frac{\sin 2\alpha }{4}\right)-2{R}_{A}^{\left(1\right)}{xR}\alpha +2{R}_{A}^{\left(1\right)}R^2\cos \alpha +R\alpha \right]}_{\alpha =0}^{\alpha =\pi +{\theta }_{1}}}\right)$$

For the geometrical parameters, we considered the values reported in Supporting Tables 1–3; these were selected to achieve a trade-off between fabrication limits, requirements of a compact design, and high performance, considering the DOE analysis results. Notably, for the simplified structure of Supporting Fig. 1c (also in green in Supporting Fig. S1b) to correctly reproduce the behavior (i.e., having an overlapping center of rotation) of the original structure (in red in Supporting Fig. S1b), we considered a curved beam with a radius 1.1 larger than that of the original structure.

### Multiphysics simulations

Coupled multiphysics simulations were performed on both the novel C-shaped and symmetrical lancet (for comparison) rotational structures using the finite element software Ansys® thermal-electric-structural interaction mode. In structural boundary conditions, actuator anchors are mechanically fixed, while all other boundaries remain free to move. For thermal boundary conditions, the faces in contact with the substrate are set at a constant temperature (22 °C). Regarding the electrical domain, a DC voltage was applied between the actuator anchors (contact pads); the MEMS rotational structure output included both the temperature and the displacement fields. When voltage is applied across the anchors, heat is generated as a result of Joule heating and simultaneously dissipated until it reaches the steady state of heat balance.

The dimensions used in the simulations are listed in Supporting Table S1. The silicon material properties used in the simulations can be found in Supporting Table S2. The lancet and symmetrical C-shaped MEMS-based rotational structures share the same width, thickness, length, and number of chevron beams in the thermal actuators, as well as the straight beam and tilted arm geometry, shuttle lengths, and rotational hinge angles.

### Device fabrication

**Fig. 1:**
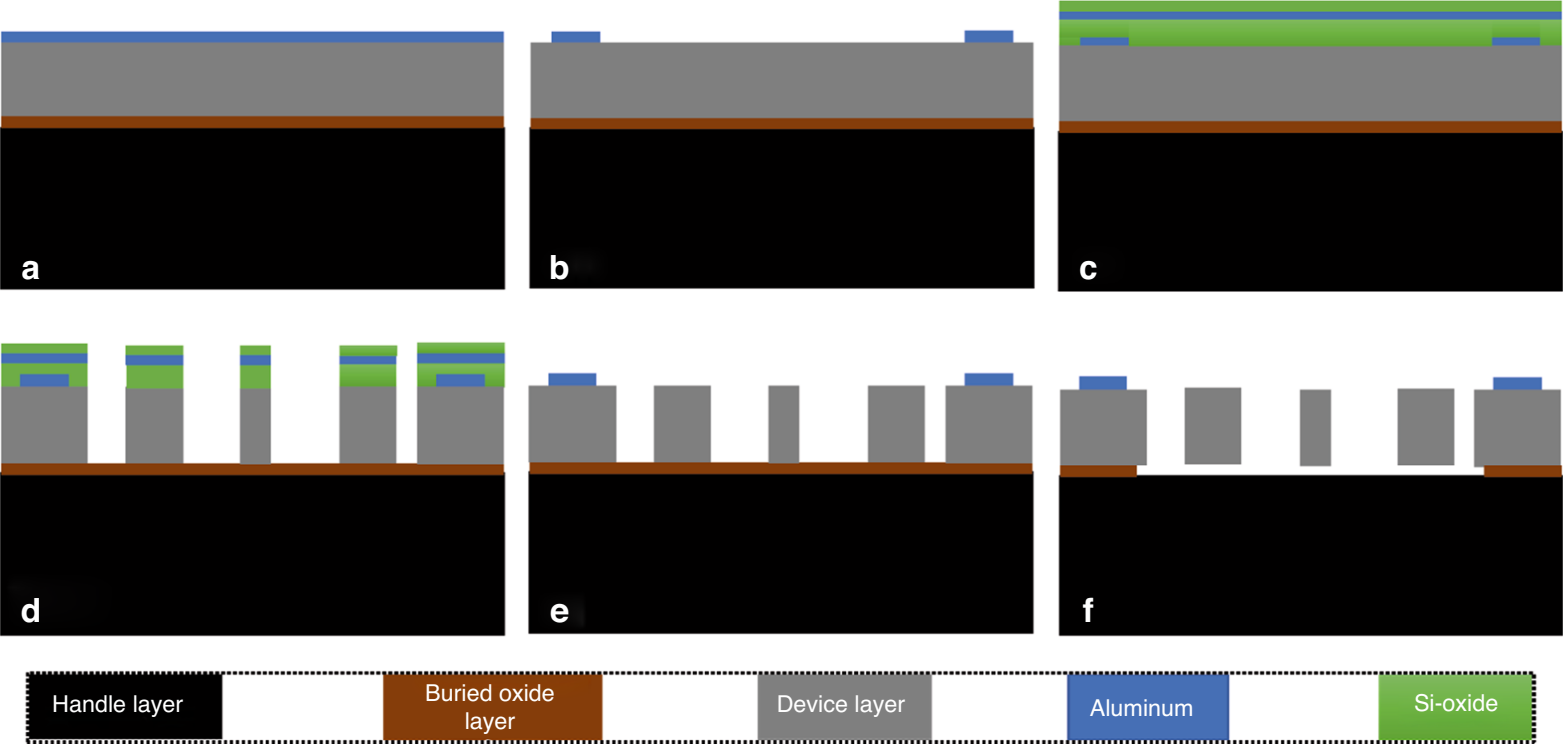
Process sequence and main steps for the fabrication of the devices. **a**, **b** Aluminum layer deposition and patterning, **c** masking layer deposition, **d** silicon structural layer etching, **e** masking layer etching, and **f** buried oxide layer etching

The devices were fabricated starting from a 6-inch (100) SOI wafer. The SOI wafer consisted of a device layer with a thickness of 25 μm, a buried SiO_2_ layer with a thickness of 1.5 μm, and a handle layer with a thickness of 525 μm. The fabrication process involved six main steps, which were similar to previous microfabrication procedures documented in^31–33^. The microfabrication process (Fig. 1) began with the cleaning of the wafer by a standard RCA (Radio Corporation of America) cleaning method to remove organic and ionic contaminants. The first and second steps involved the deposition of an aluminum layer of 800 nm thickness and patterning of the aluminum thin film to define the connection pads on the SOI wafer (Fig. 1a, b). In the third step, a 600 nm thick protective layer was deposited on top of the patterned aluminum using a PlasmaPro 100 Cobra ICP PECVD (plasma-enhanced chemical vapor deposition) deposition tool. Next, a sputtered aluminum layer with a thickness of 150 nm and a 200 nm thick silicon oxide layer were deposited as masking layers to eliminate the presence of micromasking^31^ using a PECVD process. To expose the underlying silicon, the layers were patterned with stepper photolithography and etched in a plasma etching process. The exposed silicon device layer was then etched down to the buried oxide layer using an Alcatel AMS200 DRIE (deep reactive ion etching) process, which provided anisotropic etching with higher selectivity and verticality in the fourth step to outline the main features of the device. In the fifth step, the masking layers were etched using a combination of dielectric and metal etchers to form the device. Finally, the device was released by etching the sacrificial islands and buried oxide layer with HF vapor etching using an SPTS Primaxx® uEtch etcher from SPTS Technologies Ltd.

### Experimental characterization

After fabrication, the MEMS rotational structures were characterized electromechanically using a semiconductor measurement probe station along with a Keysight B1500A semiconductor device analyzer. Four source/measure units were used to provide signal input, and the probe tips were connected to the aluminum pads on the device. Start Easy EXPERT software was used to control the measurement channel current (I)/voltage (V) sweep during the experiments. To determine the displacement of the MEMS rotational structures, the thermal actuators were actuated by applying a DC voltage ranging from 1 to 2 V, with incremental steps of 0.2 V, and the experimental displacement was measured in air by processing microphotographs captured at each voltage. To ensure that the system was in a steady state, a delay of 7 s and a hold of 2 s were implemented between each voltage increment and image capture. For each trial, offset pictures taken at 0 V were used as a reference. The experimental displacement was measured by analyzing the acquired microphotographs using the GNU Image Manipulation Program (GIMP).

## Results and discussion

**Fig. 2:**
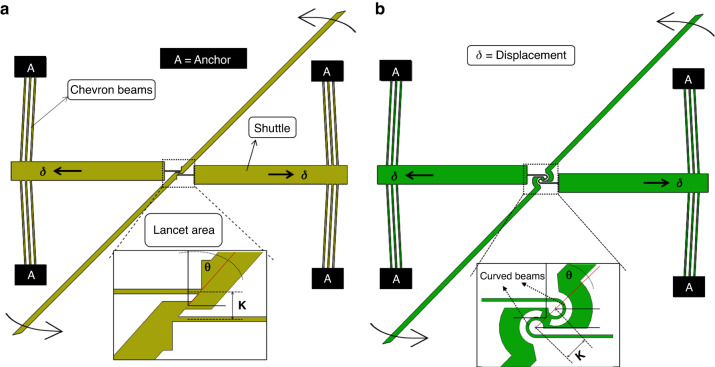
MEMS rotational structures. Schematics of the two investigated MEMS rotational structures: **a** symmetric lancet and **b** symmetrical C-shaped structures

We considered two distinct MEMS rotational structures (Fig. 2); both were designed with the same rules set, i.e., the same footprint and critical dimension. The first structure, referred to as the symmetric lancet design, was in design similar to previously published structures found in the literature^5,16,17^, but with two optimizations: the rotation points were placed along the pointer axes, and the pointer was tilted to the maximum angle (θ) compatible with the desired footprint. The second structure, the C-shaped rotational structure, was an original design novel to the field. The MEMS rotational structures depicted in Fig. 2 consisted of a series of interconnected components that worked together to produce rotational motion. These components included a double set of opposed thermal actuators consisting of chevron beams, a rotational mechanism, a straight beam, and a tilted arm. The straight beam and rotational mechanism served to facilitate movement and ensured that the tilted arm rotated smoothly and efficiently.

Both MEMS rotational structures used actuators as the source of input force and displacement. There are several driving devices available in the literature that are capable of producing displacements, including electrostatic, piezoelectric, electromagnetic, and electrothermal actuators^34^. Each of these devices has its own benefits and drawbacks. The selection of the optimal driving mechanism for actuation depends on the specific requirements of the application, including force output, displacement range, cost, and size. For example, electrostatic actuators are characterized by high force generation but limited displacement. Piezoelectric actuators provide high force density and large displacement; however, they tend to be relatively expensive. Electromagnetic actuators can produce substantial forces and displacements, but they typically require magnetic fields and may be bulky^35^. Electrothermal actuators, on the other hand, exploit the thermal expansion of materials due to electrical current flow, which results in substantial force generation and allows for actuation in multiple directions. Fast response times, high force-to-volume ratios, and low power consumption cause electrothermal actuators to be a versatile solution for various actuation applications^30^. The thermal actuator works by expanding and contracting due to changes in temperature, generating linear motion.

In our MEMS rotational structures, when a thermal actuator was activated, it produced linear motion that was transferred to the central shuttle and straight beam. The rotational mechanisms then converted this linear motion into rotational motion, which could be used for various microelectromechanical applications. The integration of thermal actuators on both sides of the central shuttle and straight beam allowed for more precise control over the rotational displacement produced by the device. Thus, MEMS rotational structures are ideal for use in microscale systems that require precise control over their motion^30^. The design of the rotational mechanism is critical to the overall performance of the rotating MEMS structure. The design parameters for the symmetric lancet MEMS rotational structure were selected based on literature^5,17,18^, while the C-shaped MEMS rotational structure was optimized using the design of experiments (DOE) method^36^ and analytical modeling. The optimization process involved systematic testing of various design combinations to determine the optimal configuration for the C-shaped structure, considering the specific requirements and constraints of the application, such as fabrication limitations.

### Design of experiments (DOE) method

The results of the DOE showed that the model was statistically significant as determined by the *p*-value, which was less than 0.05. The minimal gap between the two curved beam centers of rotation and a smaller hinge width caused the most substantial impact on the output displacements, as evidenced by the results presented in Table 2. Specifically, the results indicated that changes in the width of the curved beam (W) while holding all other parameters constant had the greatest influence on the output displacement (1st row of Table 2). Additionally, the combination of the curved beam width (W) and hinge gaps (K) exhibited a secondary influence on the performance (3rd row of Table 2). This result could be useful for optimizing the parameters of the MEMS device since it emphasizes the importance of these specific design factors in determining the overall performance of the device.

**Fig. 3:**
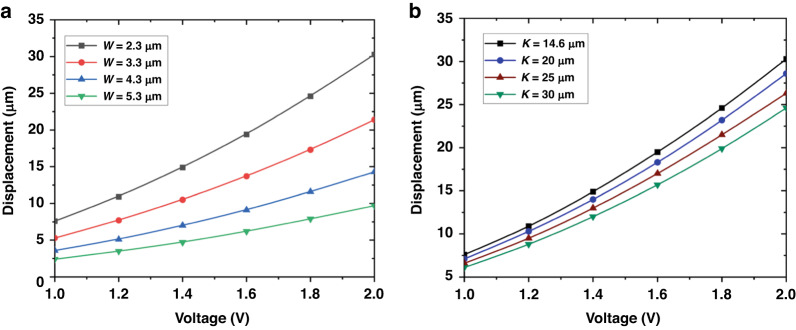
Performance analysis of the symmetrical C-shaped rotational structure. Dependence of the output displacement on **a** the curved beam width and **b** the gap between the hinges center of rotation

Based on these results, we carried out a performance analysis via Ansys® software of the symmetrical C-shaped rotational structure, examining the effects of the variations in curved beam width (Fig. 3a) and the gap between the curved beam’s center of rotation (Fig. 3b, Supporting Fig. S2). These results were consistent with the observations made from the DOE analysis provided in Table 2. Specifically, both parameters influenced the system response, and this influence was different. In particular, the displacement increased when both the width of the curved beam and the gap between the centers of rotation were reduced, which resulted in more sensitivity to a variation of the first parameter than to a variation of the second parameter. In the considered interval, the best performance was achieved when *W* = 2.3 µm and *K* = 14.6 µm, which corresponded to the values used for device fabrication.

### Analytical modeling

After substituting Eqs. (7) and (8) into Eq. (6), the strain energies of the straight and curved beams result in the following:

9 $$\begin{array}{l}{U}_{{straight}\,{beam}}=\frac{1}{2{EI}}\Bigg[\left(\frac{{x}^{3}}{3}\right)\left({\left({R}_{A}^{\left(0\right)}\right)}^{2}-2{R}_{A}^{\left(0\right)}{R}_{A}^{\left(1\right)}Y+{{R}_{A}^{\left(1\right)}}^{2}{Y}^{2}\right)\\ \qquad\qquad\qquad +\left(\frac{{x}^{2}}{2}\right)\left(2{R}_{A}^{\left(0\right)}Y-2{R}_{A}^{\left(1\right)}{Y}^{2}\right)+{Y}^{2}x\Bigg]_{x=0}^{x={x}_{s}}\end{array}$$

10 $$\begin{array}{lll}{U}_{{curved}\,{beam}}={\frac{1}{2{EI}}}\Bigg[-2Y{F}_{A}{R}^{2}\alpha \left(1+{R}_{A}^{\left(1\right)}{x}_{s}\right)+{F}_{A}{R}^{2}\sin \alpha \left(2Y+2{R}_{A}^{\left(0\right)}{x}_{s}-2{F}_{A}R\right)\\ \qquad \qquad \qquad +\,R\alpha \left(Y{R}_{A}^{\left(0\right)}{x}_{s}+{Y}^{2}{{R}_{A}^{\left(1\right)}}^{2}{{x}_{s}}^{2}-2Y{R}_{A}^{\left(0\right)}{R}_{A}^{\left(1\right)}{{x}_{s}}^{2}+{\left({R}_{A}^{\left(0\right)}\right)}^{2}{{x}_{s}}^{2}+\left({R}_{A}^{\left(0\right)}\right){x}_{s}Y\right)\\ \qquad\qquad\qquad -\,2Y{R}_{A}^{\left(0\right)}{R}^{2}\cos \alpha \left(1+2{R}_{A}^{\left(1\right)}{x}_{s}\right)+2{Y}^{2}{R}_{A}^{\left(1\right)}{R}^{2}\cos \alpha -{Y}^{2}R\alpha \left(2{R}_{A}^{\left(1\right)}{x}_{s}+1\right)\\ \qquad\qquad\qquad -\,2Y{R}_{A}^{\left(1\right)}{x}_{s}{F}_{A}{R}^{2}\sin \alpha -2Y{R}_{A}^{\left(1\right)}{R}^{2}\cos \alpha \left(Y{R}_{A}^{\left(1\right)}{x}_{s}-{F}_{A}R\right)\\ \qquad\qquad\qquad -\,{R}^{3}\left(\frac{\alpha }{2}-\frac{\sin 2\alpha}{4}\right)\left(2Y{R}_{A}^{\left(0\right)}{R}_{A}^{\left(1\right)}-{Y}^{2}{{R}_{A}^{\left(1\right)}}^{2}-{\left({R}_{A}^{\left(0\right)}\right)}^{2}\right)+2{R}_{A}^{\left(0\right)}{R}^{2}\cos \alpha \left({F}_{A}R-{R}_{A}^{\left(0\right)}{x}_{s}\right)\\ \qquad\qquad\qquad\; +\,2{R}_{A}^{\left(0\right)}{F}_{A}{R}^{3}\left(\frac{{\sin }^{2}\alpha}{2}\right)-\alpha \left(2{R}_{A}^{\left(0\right)}F{R}^{2}{x}_{s}+{{F}_{A}}^{2}{R}^{3}\right)+{{F}_{A}}^{2}{R}^{3}\left(\frac{\alpha}{2}+\frac{\sin 2\alpha}{4}\right)\Bigg]_{\alpha =0}^{\alpha =\pi +{\theta}_{1}}\end{array}$$

**Fig. 4:**
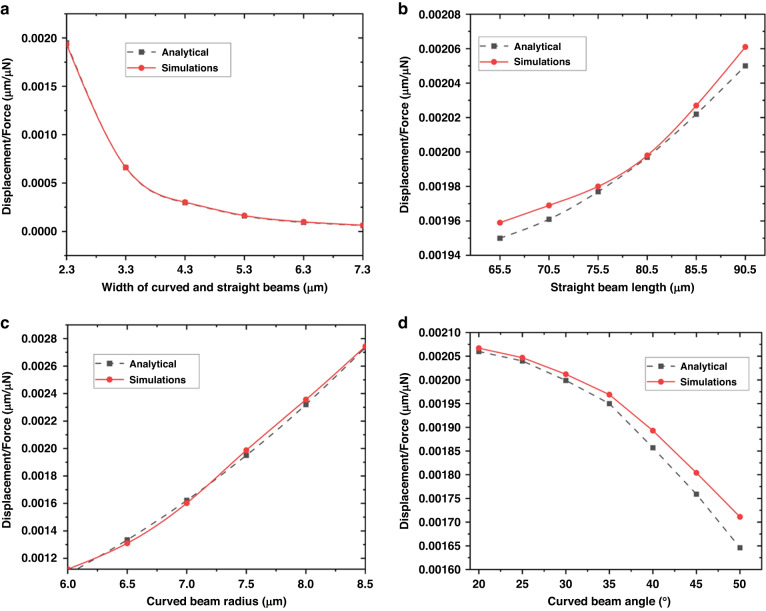
Analytical model. Investigation of the impact of the symmetrical C-shape geometry on the displacement at the beginning of the straight beam determined from the analytical model based on Castigliano’s second theorem: study of **a** curved and straight beam width, **b** straight beam length, **c** curved beam radius, and **d** angle

Supporting Fig. S3 shows the displacement *δ*_*A*_ as a function of *F*_*A*_ obtained from Eq. (5), where the strain energy was computed according to Eqs. (9) and (10), and from FEM-based structural analysis carried out through Ansys® software. As expected, the relationship *δ*_*A*_ − *F*_*A*_ was linear, with the slope according to the numerical simulations being very similar to that obtained from the analytical model (i.e., 2.6% difference). These results indicated the validity of the analytical model in predicting the behavior of the structure. By substituting Eqs. (9) and (10) in Eqs. (6) and (5), an analytical expression showing how *δ*_*A*_/*F*_*A*_ varies as a function of different geometrical quantities, such as the width and radius of the curved beam, the straight beam length, and the curved beam angle, was obtained (see Supporting Information). For example, Fig. 4a presents the relationship between *δ*_*A*_/*F*_*A*_ and the width of the curved and straight beams, as determined by the Supporting Equation (S.1). Based on these results, as the beam width increased, the displacement decreased and reached its maximum value at a beam width of 2.3 µm, as expected from the DOE results shown in the previous section.

In Fig. 4b–d, *δ*_*A*_/*F*_*A*_is presented as a function of the length of the straight beam and the radius and angle of the curved beam. The plots (based on the expressions S.2–4 in the Supporting Information) showed that the displacement had an almost linear increase as the straight beam length (especially after 70.5 µm) and radius of the curved beam were increased. However, it was significantly reduced when the angle of the curved beam increased. For comparison, in addition to the results of the analytical model, Fig. 4 shows the results obtained from FEM-based structural analysis, indicating good agreement.

### Multiphysics simulations

**Fig. 5:**
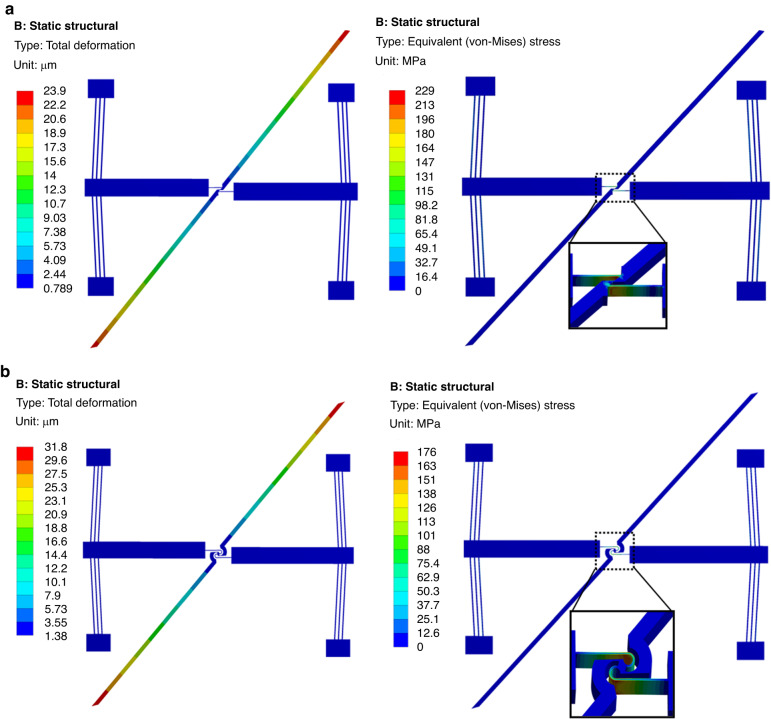
FEM simulations. Displacement and stress distribution when the actuators are biased with 2 V in symmetrical lancet (**a**) and symmetrical C-shaped (**b**) structures

Due to the heat dissipation path through the anchors in a vacuum, the highest temperatures were in the shuttle area, which was the farthest from the anchors, and resulted in nonuniform temperatures. This caused nonuniform displacement in the device as well, as shown in Fig. 5. Figure 5 also shows the stress distribution and displacement of both MEMS rotational structures under an applied voltage of 2 V. The data obtained from the simulations showed that the symmetric lancet structure exhibited a maximum displacement of 23.9 µm and a stress value of 229 MPa (Fig. 5a). On the other hand, the symmetrical C-shaped structure performed better in terms of higher output displacement (31.8 µm) and lower stress (176 MPa) at the same actuation voltage, as shown in Fig. 5b.

Finally, by using FEM-based static structural analysis, we estimated the maximum stress induced in the C-shaped beam as a function of the input displacement at the straight beam (Supporting Fig. S4).

### Experimental characterization

**Fig. 6:**
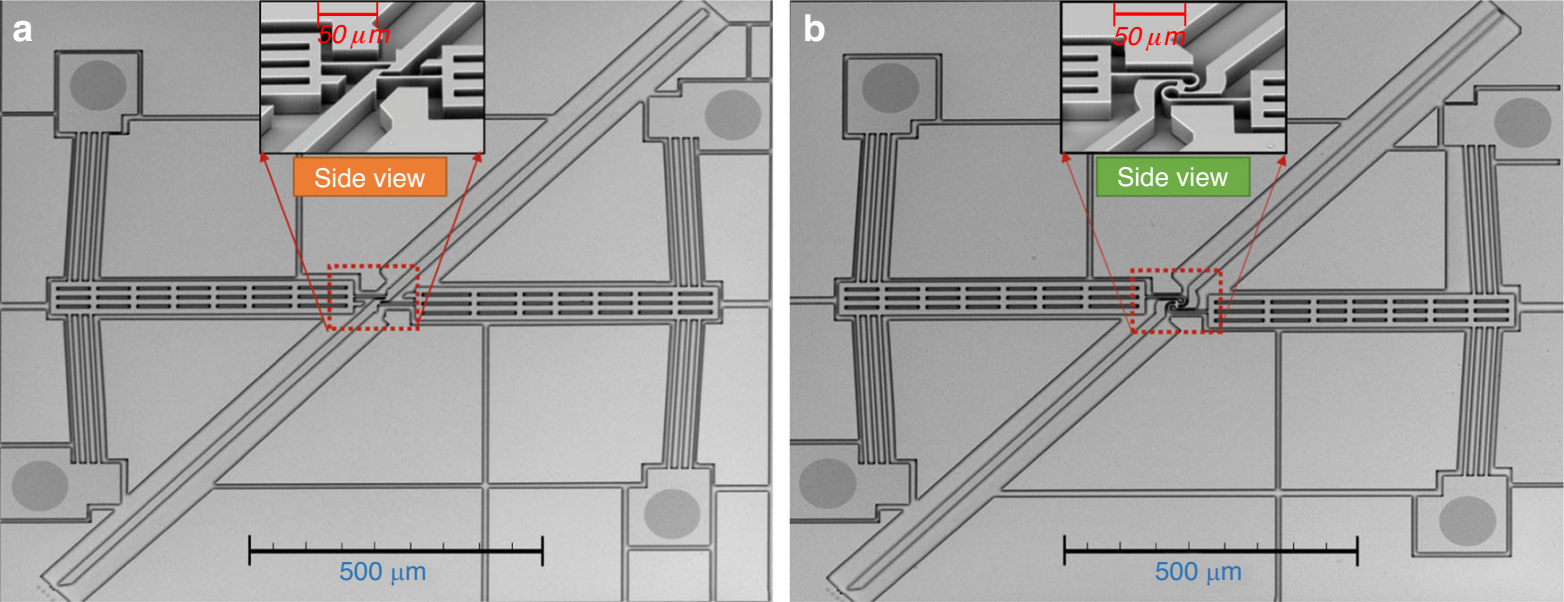
MEMS rotational structures after fabrication. SEM images of a **a** symmetric lancet and **b** symmetrical C-shaped rotational structures fabricated using SOI wafers

**Fig. 7:**
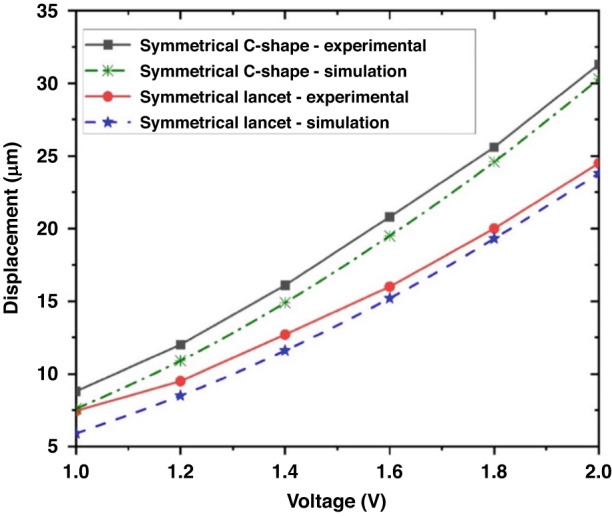
Experimental results. Comparative analysis of the symmetrical lancet and C-shaped MEMS rotational structures: simulation and experimental results

The devices were fabricated according to the process outlined in Fig. 1, and after fabrication, they appeared as shown in the scanning electron microscope (SEM) images of Fig. 6. Figure 7 shows the comparison between the displacement at the tilted arm tip of both symmetric lancet and C-shaped MEMS rotational structures using both multiphysics simulations and experimental results. Regarding the experimental and numerical results, at lower voltages, the curves started to diverge because of the fabrication tolerances, which resulted in thinner structures and increased displacement. For the C-shaped MEMS rotational structure, the difference between the experimental and simulation results was higher at lower voltages (~12%) and gradually decreased to a maximum difference of 3.3% at higher voltages. On the other hand, the symmetrical lancet structure had a maximum difference of ~2.9% at higher voltages. This discrepancy could be attributed to environmental factors, such as air resistance. When comparing the symmetrical C-shaped and lancet structures, as evidenced in the previous section, the maximum displacement of the C-shaped structure was always larger, with an increase of ~28% at 2 V compared to the symmetrical lancet model.

## Conclusions

This study contributes to the advancement of MEMS technology by introducing a novel and high-performance MEMS hinge design, which takes the shape of a symmetrical C. In this study, we showed its capability of displacement magnification when implemented in a planar rotational structure, and its performance was compared with that of a classical symmetrical lancet structure. The geometry of our proposed device was optimized based on the DOE method, which enabled the identification of the width of the C-shaped mechanism and the distance between the C beam centers of rotation; these were the geometrical parameters capable of having a significant influence on the performance (i.e., the displacement at the tip of the tilted arm) of the overall device. Experimental tests showed that our proposed device exhibited an ~28% improvement in performance compared to the symmetrical lancet structure, with the possibility of further enhancements in the future. Our analytical model based on Castigliano’s second theorem provided valuable information on the role that different geometrical parameters (e.g., angle, radius, and width of the curved beam) played in the compliance of the C-shaped mechanism and could serve as a reference point for prospective designs. Overall, the combination of our experimental, numerical, and analytical results supports the validity and accuracy of our proposed hinge design; this design can be implemented in several MEMS rotating structures, substituting standard hinges based on thin straight beams to obtain displacement magnification and thus providing a step forwards in the development of next-generation MEMS devices with improved performance and functionality.

### Supplementary information

Supporting information document
