# Supplementary material for: A C-shaped hinge for displacement magnification in MEMS rotational structures
Source: Microsyst Nanoeng. 2024 Jan 4;10:5. doi: 10.1038/s41378-023-00618-9 (PMC10764799; doi:10.1038/s41378-023-00618-9)
Supplement: Supplementary file 1 — Supporting information document [file 41378_2023_618_MOESM1_ESM.docx]

Supporting Information

**A C-shaped hinge for displacement magnification in MEMS rotational structures**

*Naga Manikanta Kommanaboina^1, 2†^, Teferi Sitotaw Yallew ^1, 2†^, Alvise Bagolini ^2*^, Maria F. Pantano ^1*^*

^1^Department of Civil, Environmental and Mechanical Engineering, University of Trento, via Mesiano, 77-38123 Trento, Italy

^2^Fondazione Bruno Kessler (FBK), Microsystems Technology (MST), via S. Croce, 77-38122 Trento, Italy

*Correspondence: [maria.pantano@unitn.it](mailto:maria.pantano@unitn.it), [bagolini@fbk.eu](mailto:bagolini@fbk.eu)

^†^: Equal contribution


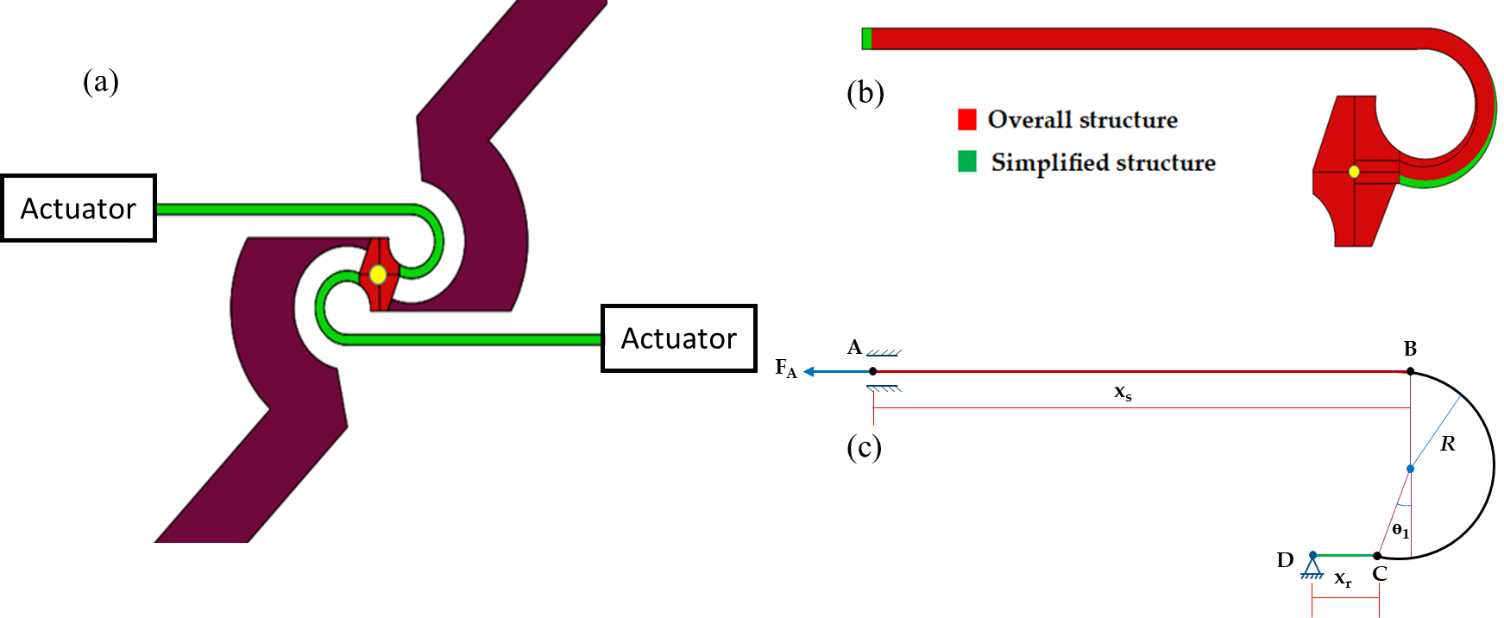


Figure S1: Schematic diagram of symmetrical C-shaped structure implemented in the real device (a), comparison between the designed and simplified curved beam structure (b) studied in the analytical model (c).


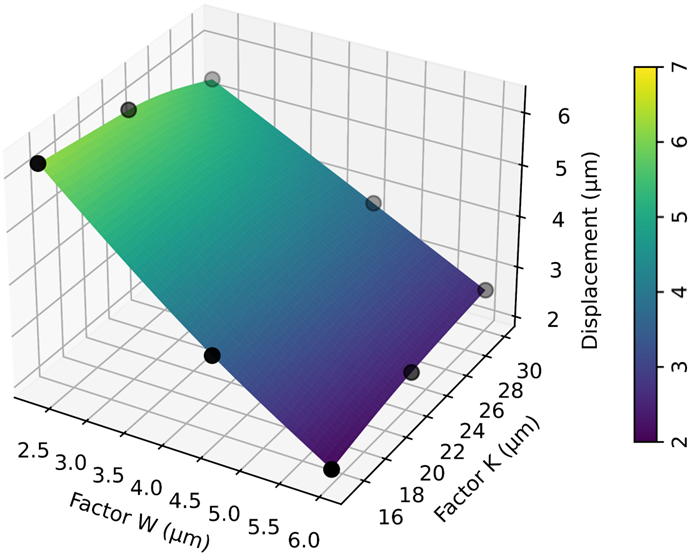


Figure S2: Optimizing variable performance through 3D response surface analysis of symmetrical C-shaped rotational structure.


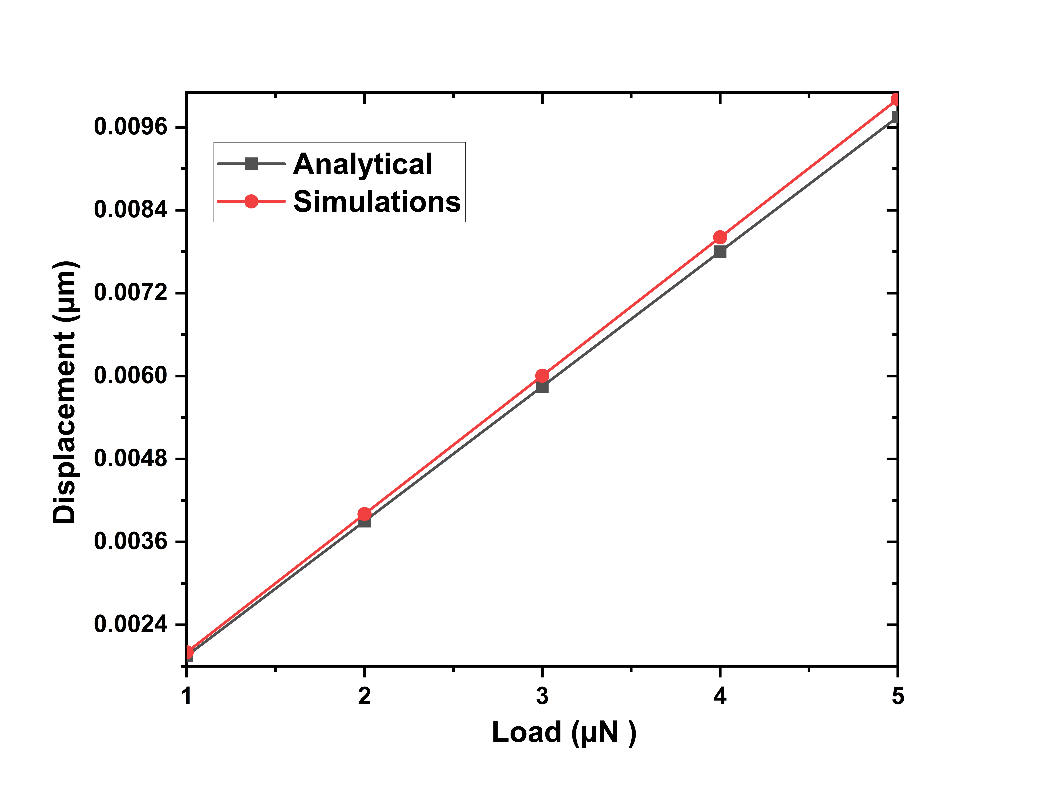


Figure S3: Comparison between the horizontal displacement at the beginning of straight beam obtained from analytical modeling and numerical simulations as a function of the force delivered by the thermal actuators.


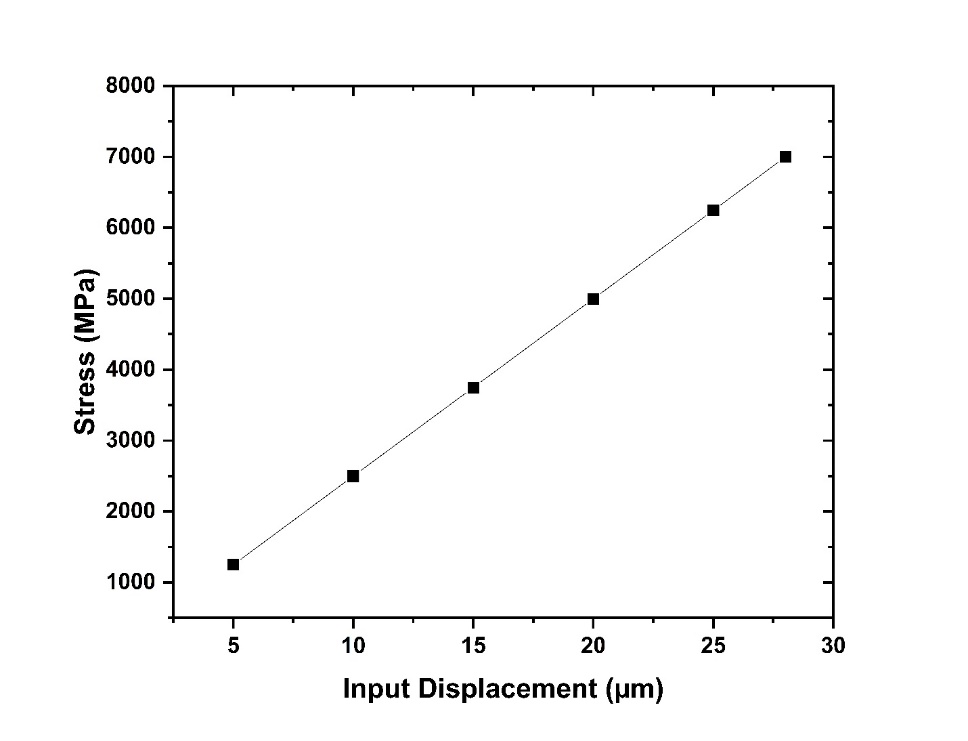


Figure S4: Stress versus displacement of symmetrical C-shaped rotational structure.

Table S1. Geometrical parameters of MEMS rotational structures

| Actuator beam length | 300 µm |
| --- | --- |
| Actuator beam width | 4.7 µm |
| Actuator beam angle | 3° |
| Total number of beams | 6 |
| Device thickness | 25 µm |
| Straight beam length | 65.5 µm |
| Straight beam width | 2.3 µm |
| Curved beam width | 2.3 µm |
| Curved beam radius (R) | 7.5 µm |
| Curved beam angle (θ) | 34.15° |
| Gap (K) | 14.6 µm |
| Tilted arm length | 780 µm |
| Tilted arm width | 16.7 µm |

Tale S2. Silicon properties [21].

| Young’s modulus (E) | 169 GPa |
| --- | --- |
| Poisson's ratio (ν) | 0.28 |
| Density (ρ) | 2330 kg/m^3^ |
| Thermal conductivity (K) | 130 [W/(m^.^K)] |
| Coefficient of thermal expansion (α) | 2.6 × 10^-6^ K^-1^ |
| Resistivity | 0.005 Ω^.^cm |

Table S3. Geometrical parameters of curved beam.

| Angle ($\theta_{1})$ | $34.15^{\circ}$ |
| --- | --- |
| Curved beam radius (R) | $7.5 \mu m$ |
| $W$idth (W) | $2.3 \mu m$ |
| Straight beam length$(x_{s})$ | 65.5 $\mu m$ |
| Thickness (h) | 25 $\mu m$ |
| Rigid beam length$(x_{r})$ | 5.3 $\mu m$ |

By computing the derivative of the strain energy reported in equation (5) with respect to the *F_A_*, it is possible to find the displacement at the beginning of the straight beam $\delta_{A}$ as a function of different geometrical parameters. In particular, the following equations ((S.1) to (S.4)), report $\delta_{A}/F_{A}$ as a function of the width of the straight and curved beams, radius of the curved beam, length of the straight beam and angle of the curved beam, respectively. These relationships together with the geometrical parameters reported in Table S3 were used to draw the plots reported in Figure 4.

${\frac{\delta_{A}}{F_{A}}(W)= \left( \frac{6}{hEW^{3}} \right)\left[ 2\left( \frac{R\left( 1+cos \theta_{1} \right)}{x_{s}-(x_{r}+R\sin\theta_{1})} \right)^{2}\left( \frac{x^{3}}{3} \right)+11.0104\left( \frac{R\left( 1+cos \theta_{1} \right)}{x_{s}-\left( x_{r}+R\sin\theta_{1} \right)} \right)\left( \frac{1}{x_{s}-\left( x_{r}+R\sin\theta_{1} \right)} \right)\left( \frac{x^{3}}{3} \right)-11.0104\left( \frac{R\left( 1+cos \theta_{1} \right)}{x_{s}-\left( x_{r}+R\sin\theta_{1} \right)} \right)\left( \frac{x^{2}}{2} \right)+15.1536\left( \frac{1}{x_{s}-\left( x_{r}+R\sin\theta_{1} \right)} \right)^{2}\left( \frac{x^{3}}{3} \right)-30.3072\left( \frac{1}{x_{s}-\left( x_{r}+R\sin\theta_{1} \right)} \right)\left( \frac{x^{2}}{2} \right)+15.1536 x \right]}_{x=0}^{x=x_{s}}+{\left( \frac{6}{hEW^{3}} \right)\left[ 11.0104 R^{2}\alpha-11.0104 R^{2}sin \alpha-5.5052\left( \frac{R\left( 1+cos \theta_{1} \right)}{x_{s}-\left( x_{r}+R\sin\theta_{1} \right)} \right)x_{s}R\alpha+11.0104 \left( \frac{R\left( 1+cos \theta_{1} \right)}{x_{s}-\left( x_{r}+R\sin\theta_{1} \right)} \right)R^{2}cos \alpha+30.3072 \left( \frac{1}{x_{s}-\left( x_{r}+R\sin\theta_{1} \right)} \right)R^{2}cos \alpha-30.3072 \left( \frac{1}{x_{s}-\left( x_{r}+R\sin\theta_{1} \right)} \right)x_{s}R\alpha-5.5052 R\alpha-11.0104 \left( \frac{1}{x_{s}-\left( x_{r}+R\sin\theta_{1} \right)} \right)x_{s}R^{2}\alpha+11.0104 \left( \frac{1}{x_{s}-\left( x_{r}+R\sin\theta_{1} \right)} \right)x_{s}R^{2}sin \alpha+11.0104\left( \frac{R\left( 1+cos \theta_{1} \right)}{x_{s}-\left( x_{r}+R\sin\theta_{1} \right)} \right)\left( \frac{1}{x_{s}-\left( x_{r}+R\sin\theta_{1} \right)} \right){x_{s}}^{2}R\alpha-22.0208\left( \frac{R\left( 1+cos \theta_{1} \right)}{x_{s}-\left( x_{r}+R\sin\theta_{1} \right)} \right)\left( \frac{1}{x_{s}-\left( x_{r}+R\sin\theta_{1} \right)} \right)x_{s}R^{2}cos \alpha-30.3072 \left( \frac{1}{x_{s}-\left( x_{r}+R\sin\theta_{1} \right)} \right)^{2}x_{s}R^{2}cos \alpha+15.1536 \left( \frac{1}{x_{s}-\left( x_{r}+R\sin\theta_{1} \right)} \right)^{2}{x_{s}}^{2}R\alpha+11.0104 \left( \frac{1}{x_{s}-\left( x_{r}+R\sin\theta_{1} \right)} \right)R^{3}cos \alpha+11.0104\left( \frac{R\left( 1+cos \theta_{1} \right)}{x_{s}-\left( x_{r}+R\sin\theta_{1} \right)} \right)\left( \frac{1}{x_{s}-\left( x_{r}+R\sin\theta_{1} \right)} \right)R^{3}\left( \frac{\alpha}{2}-\frac{\sin2\alpha}{4} \right)+15.1536 \left( \frac{1}{x_{s}-\left( x_{r}+R\sin\theta_{1} \right)} \right)^{2}R^{3}\left( \frac{\alpha}{2}-\frac{\sin2\alpha}{4} \right)+ 4\left( \frac{R\left( 1+cos \theta_{1} \right)}{x_{s}-\left( x_{r}+R\sin\theta_{1} \right)} \right)R^{3}cos \alpha+ 4\left( \frac{R\left( 1+cos \theta_{1} \right)}{x_{s}-\left( x_{r}+R\sin\theta_{1} \right)} \right)R^{3}\left( \frac{{sin}^{2}\alpha}{2} \right)- 4\left( \frac{R\left( 1+cos \theta_{1} \right)}{x_{s}-\left( x_{r}+R\sin\theta_{1} \right)} \right)^{2}R^{2}x_{s} cos \alpha+2\left( \frac{R\left( 1+cos \theta_{1} \right)}{x_{s}-\left( x_{r}+R\sin\theta_{1} \right)} \right)^{2}R^{3}\left( \frac{\alpha}{2}-\frac{\sin2\alpha}{4} \right)- 4\left( \frac{R\left( 1+cos \theta_{1} \right)}{x_{s}-\left( x_{r}+R\sin\theta_{1} \right)} \right)R^{2}x_{s}\alpha+ 4\left( \frac{R\left( 1+cos \theta_{1} \right)}{x_{s}-\left( x_{r}+R\sin\theta_{1} \right)} \right)R^{2}x_{s} sin \alpha+ 2\left( \frac{R\left( 1+cos \theta_{1} \right)}{x_{s}-\left( x_{r}+R\sin\theta_{1} \right)} \right)^{2}{x_{s}}^{2}R\alpha-5.5052\left( \frac{R\left( 1+cos \theta_{1} \right)}{x_{s}-(x_{r}+R\sin\theta_{1})} \right)x_{s}R\alpha- 4R^{3}sin \alpha+ 2R^{3}\left( \frac{\alpha}{2}+\frac{\sin2\alpha}{4} \right)+ 2R^{3}\alpha\right]}_{\alpha=0^{\circ}}^{\alpha=214.15^{\circ}}$ (S.1)

${\frac{\delta_{A}}{F_{A}}(R)= \left( \frac{1}{2EI} \right)\left[ 2\left( \frac{R\left( 1+cos \theta_{1} \right)}{x_{s}-(x_{r}+R\sin\theta_{1})} \right)^{2}\left( \frac{x^{3}}{3} \right)-4\left( Y^{'} \right)\left( \frac{R\left( 1+cos \theta_{1} \right)}{x_{s}-\left( x_{r}+R\sin\theta_{1} \right)} \right)\left( \frac{1}{x_{s}-\left( x_{r}+R\sin\theta_{1} \right)} \right)\left( \frac{x^{3}}{3} \right)+4\left( Y^{'} \right)\left( \frac{R\left( 1+cos \theta_{1} \right)}{x_{s}-\left( x_{r}+R\sin\theta_{1} \right)} \right)\left( \frac{x^{2}}{2} \right)+2\left( Y^{'} \right)^{2}\left( \frac{1}{x_{s}-\left( x_{r}+R\sin\theta_{1} \right)} \right)^{2}\left( \frac{x^{3}}{3} \right)-4\left( Y^{'} \right)^{2}\left( \frac{1}{x_{s}-\left( x_{r}+R\sin\theta_{1} \right)} \right)\left( \frac{x^{2}}{2} \right)+2\left( Y^{'} \right)^{2} x \right]}_{x=0}^{x=x_{s}}+{\left( \frac{1}{2EI} \right)\left[ - 4 \left( Y^{'} \right)R^{2}\alpha+4 \left( Y^{'} \right) R^{2}sin \alpha+2\left( Y^{'} \right)\left( \frac{R\left( 1+cos \theta_{1} \right)}{x_{s}-\left( x_{r}+R\sin\theta_{1} \right)} \right)x_{s}R\alpha-4 \left( Y^{'} \right) \left( \frac{R\left( 1+cos \theta_{1} \right)}{x_{s}-\left( x_{r}+R\sin\theta_{1} \right)} \right)R^{2}cos \alpha+4\left( Y^{'} \right)^{2} \left( \frac{1}{x_{s}-\left( x_{r}+R\sin\theta_{1} \right)} \right)R^{2}cos \alpha-4\left( Y^{'} \right)^{2} \left( \frac{1}{x_{s}-\left( x_{r}+R\sin\theta_{1} \right)} \right)x_{s}R\alpha+2\left( Y^{'} \right) R\alpha+4\left( Y^{'} \right) \left( \frac{1}{x_{s}-\left( x_{r}+R\sin\theta_{1} \right)} \right)x_{s}R^{2}\alpha-4 \left( Y^{'} \right) \left( \frac{1}{x_{s}-\left( x_{r}+R\sin\theta_{1} \right)} \right)x_{s}R^{2}sin \alpha-4 \left( Y^{'} \right)\left( \frac{R\left( 1+cos \theta_{1} \right)}{x_{s}-\left( x_{r}+R\sin\theta_{1} \right)} \right)\left( \frac{1}{x_{s}-\left( x_{r}+R\sin\theta_{1} \right)} \right){x_{s}}^{2}R\alpha+8 \left( Y^{'} \right)\left( \frac{R\left( 1+cos \theta_{1} \right)}{x_{s}-\left( x_{r}+R\sin\theta_{1} \right)} \right)\left( \frac{1}{x_{s}-\left( x_{r}+R\sin\theta_{1} \right)} \right)x_{s}R^{2}cos \alpha-4\left( Y^{'} \right)^{2} \left( \frac{1}{x_{s}-\left( x_{r}+R\sin\theta_{1} \right)} \right)^{2}x_{s}R^{2}cos \alpha+2\left( Y^{'} \right)^{2} \left( \frac{1}{x_{s}-\left( x_{r}+R\sin\theta_{1} \right)} \right)^{2}{x_{s}}^{2}R\alpha-4 \left( Y^{'} \right) \left( \frac{1}{x_{s}-\left( x_{r}+R\sin\theta_{1} \right)} \right)R^{3}cos \alpha-4 \left( Y^{'} \right)\left( \frac{R\left( 1+cos \theta_{1} \right)}{x_{s}-\left( x_{r}+R\sin\theta_{1} \right)} \right)\left( \frac{1}{x_{s}-\left( x_{r}+R\sin\theta_{1} \right)} \right)R^{3}\left( \frac{\alpha}{2}-\frac{\sin2\alpha}{4} \right)+2\left( Y^{'} \right)^{2} \left( \frac{1}{x_{s}-\left( x_{r}+R\sin\theta_{1} \right)} \right)^{2}R^{3}\left( \frac{\alpha}{2}-\frac{\sin2\alpha}{4} \right)+ 4\left( \frac{R\left( 1+cos \theta_{1} \right)}{x_{s}-\left( x_{r}+R\sin\theta_{1} \right)} \right)R^{3}cos \alpha+ 4\left( \frac{R\left( 1+cos \theta_{1} \right)}{x_{s}-\left( x_{r}+R\sin\theta_{1} \right)} \right)R^{3}\left( \frac{{sin}^{2}\alpha}{2} \right)- 4\left( \frac{R\left( 1+cos \theta_{1} \right)}{x_{s}-\left( x_{r}+R\sin\theta_{1} \right)} \right)^{2}R^{2}x_{s} cos \alpha+2\left( \frac{R\left( 1+cos \theta_{1} \right)}{x_{s}-\left( x_{r}+R\sin\theta_{1} \right)} \right)^{2}R^{3}\left( \frac{\alpha}{2}-\frac{\sin2\alpha}{4} \right)- 4\left( \frac{R\left( 1+cos \theta_{1} \right)}{x_{s}-\left( x_{r}+R\sin\theta_{1} \right)} \right)R^{2}x_{s}\alpha+ 4\left( \frac{R\left( 1+cos \theta_{1} \right)}{x_{s}-\left( x_{r}+R\sin\theta_{1} \right)} \right)R^{2}x_{s} sin \alpha+ 2\left( \frac{R\left( 1+cos \theta_{1} \right)}{x_{s}-\left( x_{r}+R\sin\theta_{1} \right)} \right)^{2}{x_{s}}^{2}R\alpha+2\left( Y^{'} \right)\left( \frac{R\left( 1+cos \theta_{1} \right)}{x_{s}-(x_{r}+R\sin\theta_{1})} \right)x_{s}R\alpha- 4R^{3}sin \alpha+ 2R^{3}\left( \frac{\alpha}{2}+\frac{\sin2\alpha}{4} \right)+ 2R^{3}\alpha\right]}_{\alpha=0^{\circ}}^{\alpha=214.15^{\circ}}$ (S.2)

${\frac{\delta_{A}}{F_{A}}(x)= \left( \frac{1}{2EI} \right)\left[ 2\left( \frac{R\left( 1+cos \theta_{1} \right)}{x-(x_{r}+R\sin\theta_{1})} \right)^{2}\left( \frac{x^{3}}{3} \right)-4\left( Y^{'} \right)\left( \frac{R\left( 1+cos \theta_{1} \right)}{x-\left( x_{r}+R\sin\theta_{1} \right)} \right)\left( \frac{1}{x-\left( x_{r}+R\sin\theta_{1} \right)} \right)\left( \frac{x^{3}}{3} \right)+4\left( Y^{'} \right)\left( \frac{R\left( 1+cos \theta_{1} \right)}{x-\left( x_{r}+R\sin\theta_{1} \right)} \right)\left( \frac{x^{2}}{2} \right)+2\left( Y^{'} \right)^{2}\left( \frac{1}{x-\left( x_{r}+R\sin\theta_{1} \right)} \right)^{2}\left( \frac{x^{3}}{3} \right)-4\left( Y^{'} \right)^{2}\left( \frac{1}{x-\left( x_{r}+R\sin\theta_{1} \right)} \right)\left( \frac{x^{2}}{2} \right)+2\left( Y^{'} \right)^{2} x \right]}_{0}^{x}+{\left( \frac{1}{2EI} \right)\left[ - 4 \left( Y^{'} \right)R^{2}\alpha+4 \left( Y^{'} \right) R^{2}sin \alpha+2\left( Y^{'} \right)\left( \frac{R\left( 1+cos \theta_{1} \right)}{x-\left( x_{r}+R\sin\theta_{1} \right)} \right)xR\alpha-4 \left( Y^{'} \right) \left( \frac{R\left( 1+cos \theta_{1} \right)}{x-(x_{r}+R\sin\theta_{1})} \right)R^{2}cos \alpha+4\left( Y^{'} \right)^{2} \left( \frac{1}{x-\left( x_{r}+R\sin\theta_{1} \right)} \right)R^{2}cos \alpha-4\left( Y^{'} \right)^{2} \left( \frac{1}{x-\left( x_{r}+R\sin\theta_{1} \right)} \right)xR\alpha+2\left( Y^{'} \right) R\alpha+4\left( Y^{'} \right) \left( \frac{1}{x-\left( x_{r}+R\sin\theta_{1} \right)} \right)xR^{2}\alpha-4 \left( Y^{'} \right) \left( \frac{1}{x-\left( x_{r}+R\sin\theta_{1} \right)} \right)xR^{2}sin \alpha-4 \left( Y^{'} \right)\left( \frac{R\left( 1+cos \theta_{1} \right)}{x-(x_{r}+R\sin\theta_{1})} \right)\left( \frac{1}{x-\left( x_{r}+R\sin\theta_{1} \right)} \right)x^{2}R\alpha+8 \left( Y^{'} \right)\left( \frac{R\left( 1+cos \theta_{1} \right)}{x-(x_{r}+R\sin\theta_{1})} \right)\left( \frac{1}{x-\left( x_{r}+R\sin\theta_{1} \right)} \right)xR^{2}cos \alpha-4\left( Y^{'} \right)^{2} \left( \frac{1}{x-\left( x_{r}+R\sin\theta_{1} \right)} \right)^{2}xR^{2}cos \alpha+2\left( Y^{'} \right)^{2} \left( \frac{1}{x-\left( x_{r}+R\sin\theta_{1} \right)} \right)^{2}x^{2}R\alpha-4 \left( Y^{'} \right) \left( \frac{1}{x-\left( x_{r}+R\sin\theta_{1} \right)} \right)R^{3}cos \alpha-4 \left( Y^{'} \right)\left( \frac{R\left( 1+cos \theta_{1} \right)}{x-(x_{r}+R\sin\theta_{1})} \right)\left( \frac{1}{x-\left( x_{r}+R\sin\theta_{1} \right)} \right)R^{3}\left( \frac{\alpha}{2}-\frac{\sin2\alpha}{4} \right)+2\left( Y^{'} \right)^{2} \left( \frac{1}{x-\left( x_{r}+R\sin\theta_{1} \right)} \right)^{2}R^{3}\left( \frac{\alpha}{2}-\frac{\sin2\alpha}{4} \right)+ 4\left( \frac{R\left( 1+cos \theta_{1} \right)}{x-(x_{r}+R\sin\theta_{1})} \right)R^{3}cos \alpha+ 4\left( \frac{R\left( 1+cos \theta_{1} \right)}{x-(x_{r}+R\sin\theta_{1})} \right)R^{3}\left( \frac{{sin}^{2}\alpha}{2} \right)- 4\left( \frac{R\left( 1+cos \theta_{1} \right)}{x-(x_{r}+R\sin\theta_{1})} \right)^{2}R^{2}x cos \alpha+2\left( \frac{R\left( 1+cos \theta_{1} \right)}{x-(x_{r}+R\sin\theta_{1})} \right)^{2}R^{3}\left( \frac{\alpha}{2}-\frac{\sin2\alpha}{4} \right)- 4\left( \frac{R\left( 1+cos \theta_{1} \right)}{x-(x_{r}+R\sin\theta_{1})} \right)R^{2}x\alpha+ 4\left( \frac{R\left( 1+cos \theta_{1} \right)}{x-(x_{r}+R\sin\theta_{1})} \right)R^{2}x sin \alpha+ 2\left( \frac{R\left( 1+cos \theta_{1} \right)}{x-(x_{r}+R\sin\theta_{1})} \right)^{2}x^{2}R\alpha+2\left( Y^{'} \right)\left( \frac{R\left( 1+cos \theta_{1} \right)}{x-(x_{r}+R\sin\theta_{1})} \right)xR\alpha- 4R^{3}sin \alpha+ 2R^{3}\left( \frac{\alpha}{2}+\frac{\sin2\alpha}{4} \right)+ 2R^{3}\alpha\right]}_{\alpha=0^{\circ}}^{\alpha=214.15^{\circ}}$ (S.3)

${\frac{\delta_{A}}{F_{A}}(\theta_{1})= \left( \frac{1}{2EI} \right)\left[ 2\left( \frac{R\left( 1+cos \theta_{1} \right)}{x_{s}-(x_{r}+R\sin\theta_{1})} \right)^{2}\left( \frac{x^{3}}{3} \right)-4\left( Y^{'} \right)\left( \frac{R\left( 1+cos \theta_{1} \right)}{x_{s}-\left( x_{r}+R\sin\theta_{1} \right)} \right)\left( \frac{1}{x_{s}-\left( x_{r}+R\sin\theta_{1} \right)} \right)\left( \frac{x^{3}}{3} \right)+4\left( Y^{'} \right)\left( \frac{R\left( 1+cos \theta_{1} \right)}{x_{s}-\left( x_{r}+R\sin\theta_{1} \right)} \right)\left( \frac{x^{2}}{2} \right)+2\left( Y^{'} \right)^{2}\left( \frac{1}{x_{s}-\left( x_{r}+R\sin\theta_{1} \right)} \right)^{2}\left( \frac{x^{3}}{3} \right)-4\left( Y^{'} \right)^{2}\left( \frac{1}{x_{s}-\left( x_{r}+R\sin\theta_{1} \right)} \right)\left( \frac{x^{2}}{2} \right)+2\left( Y^{'} \right)^{2} x \right]}_{x=0}^{x=x_{s}}+{\left( \frac{1}{2EI} \right)\left[ - 4 \left( Y^{'} \right)R^{2}\alpha+4 \left( Y^{'} \right) R^{2}sin \alpha+2\left( Y^{'} \right)\left( \frac{R\left( 1+cos \theta_{1} \right)}{x_{s}-\left( x_{r}+R\sin\theta_{1} \right)} \right)x_{s}R\alpha-4 \left( Y^{'} \right) \left( \frac{R\left( 1+cos \theta_{1} \right)}{x_{s}-\left( x_{r}+R\sin\theta_{1} \right)} \right)R^{2}cos \alpha+4\left( Y^{'} \right)^{2} \left( \frac{1}{x_{s}-\left( x_{r}+R\sin\theta_{1} \right)} \right)R^{2}cos \alpha-4\left( Y^{'} \right)^{2} \left( \frac{1}{x_{s}-\left( x_{r}+R\sin\theta_{1} \right)} \right)x_{s}R\alpha+2\left( Y^{'} \right) R\alpha+4\left( Y^{'} \right) \left( \frac{1}{x_{s}-\left( x_{r}+R\sin\theta_{1} \right)} \right)x_{s}R^{2}\alpha-4 \left( Y^{'} \right) \left( \frac{1}{x_{s}-\left( x_{r}+R\sin\theta_{1} \right)} \right)x_{s}R^{2}sin \alpha-4 \left( Y^{'} \right)\left( \frac{R\left( 1+cos \theta_{1} \right)}{x_{s}-\left( x_{r}+R\sin\theta_{1} \right)} \right)\left( \frac{1}{x_{s}-\left( x_{r}+R\sin\theta_{1} \right)} \right){x_{s}}^{2}R\alpha+8 \left( Y^{'} \right)\left( \frac{R\left( 1+cos \theta_{1} \right)}{x_{s}-\left( x_{r}+R\sin\theta_{1} \right)} \right)\left( \frac{1}{x_{s}-\left( x_{r}+R\sin\theta_{1} \right)} \right)x_{s}R^{2}\cos\alpha-4\left( Y^{'} \right)^{2} \left( \frac{1}{x_{s}-\left( x_{r}+R\sin\theta_{1} \right)} \right)^{2}x_{s}R^{2}\cos\alpha+2\left( Y^{'} \right)^{2} \left( \frac{1}{x_{s}-\left( x_{r}+R\sin\theta_{1} \right)} \right)^{2}{x_{s}}^{2}R\alpha-4 \left( Y^{'} \right) \left( \frac{1}{x_{s}-\left( x_{r}+R\sin\theta_{1} \right)} \right)R^{3}cos \alpha-4 \left( Y^{'} \right)\left( \frac{R\left( 1+cos \theta_{1} \right)}{x_{s}-\left( x_{r}+R\sin\theta_{1} \right)} \right)\left( \frac{1}{x_{s}-\left( x_{r}+R\sin\theta_{1} \right)} \right)R^{3}\left( \frac{\alpha}{2}-\frac{\sin2\alpha}{4} \right)+2\left( Y^{'} \right)^{2} \left( \frac{1}{x_{s}-\left( x_{r}+R\sin\theta_{1} \right)} \right)^{2}R^{3}\left( \frac{\alpha}{2}-\frac{\sin2\alpha}{4} \right)+ 4\left( \frac{R\left( 1+cos \theta_{1} \right)}{x_{s}-\left( x_{r}+R\sin\theta_{1} \right)} \right)R^{3}cos \alpha+ 4\left( \frac{R\left( 1+cos \theta_{1} \right)}{x_{s}-\left( x_{r}+R\sin\theta_{1} \right)} \right)R^{3}\left( \frac{{sin}^{2}\alpha}{2} \right)- 4\left( \frac{R\left( 1+cos \theta_{1} \right)}{x_{s}-\left( x_{r}+R\sin\theta_{1} \right)} \right)^{2}R^{2}x_{s} cos \alpha+2\left( \frac{R\left( 1+cos \theta_{1} \right)}{x_{s}-\left( x_{r}+R\sin\theta_{1} \right)} \right)^{2}R^{3}\left( \frac{\alpha}{2}-\frac{\sin2\alpha}{4} \right)- 4\left( \frac{R\left( 1+cos \theta_{1} \right)}{x_{s}-\left( x_{r}+R\sin\theta_{1} \right)} \right)R^{2}x_{s}\alpha+ 4\left( \frac{R\left( 1+cos \theta_{1} \right)}{x_{s}-\left( x_{r}+R\sin\theta_{1} \right)} \right)R^{2}x_{s} sin \alpha+ 2\left( \frac{R\left( 1+cos \theta_{1} \right)}{x_{s}-\left( x_{r}+R\sin\theta_{1} \right)} \right)^{2}{x_{s}}^{2}R\alpha+2\left( Y^{'} \right)\left( \frac{R\left( 1+cos \theta_{1} \right)}{x_{s}-(x_{r}+R\sin\theta_{1})} \right)x_{s}R\alpha- 4R^{3}sin \alpha+ 2R^{3}\left( \frac{\alpha}{2}+\frac{\sin2\alpha}{4} \right)+ 2R^{3}\alpha\right]}_{0^{\circ}}^{\pi+\theta_{1}}$ (S.4)

Where $Y^{'}$ is the moment at the slider, normalized by the force, $F_{A}$.
